# Resistance Training and Testosterone Levels in Male Patients with Chronic Kidney Disease Undergoing Dialysis

**DOI:** 10.1155/2014/121273

**Published:** 2014-04-03

**Authors:** Stig Molsted, Jesper L. Andersen, Inge Eidemak, Adrian P. Harrison, Niels Jørgensen

**Affiliations:** ^1^Department of Cardiology, Nephrology & Endocrinology, Nordsjællands University Hospital, Dyrehavevej 29, Opg. 52C Plan 4, 3400 Hillerød, Denmark; ^2^Institute of Sports Medicine Copenhagen, Bispebjerg University Hospital, Bispebjerg Bakke 23, 2400 Copenhagen NV, Denmark; ^3^Department of Nephrology P, Rigshospitalet, Copenhagen University Hospital, Blegdamsvej 9, 2100 Copenhagen Ø, Denmark; ^4^Department of Veterinary Clinical and Animal Sciences, Faculty of Health and Medical Sciences, Copenhagen University, Grønnegårdsvej 15, 1870 Frederiksberg C, Denmark; ^5^University Department of Growth and Reproduction, Rigshospitalet, Copenhagen University Hospital, Blegdamsvej 9, 2100 Copenhagen Ø, Denmark

## Abstract

*Background.* We investigated serum testosterone and insulin-like growth factor 1 (IGF-1) levels' associations with muscle fibre size and resistance training in male dialysis patients.* Methods.* Male patients were included in a 16-week control period followed by 16 weeks of resistance training thrice weekly. Blood samples were obtained to analyse testosterone, luteinizing hormone (LH), IGF-1, and IGF-binding protein 3. Muscle fibres' size was analysed in biopsies from* m. vastus lateralis*.* Results.* The patients' testosterone levels were within the normal range at baseline (*n* = 20) (19.5 (8.2–52.1) nmol/L versus 17.6 (16.1–18.0), resp.) whereas LH levels were higher (13.0 (5.5–82.8) U/L versus 4.3 (3.3–4.6), *P* < 0.001, resp.). IGF-1 and IGF-binding protein 3 levels were higher in the patients compared with reference values (203 (59–590) ng/mL versus 151 (128–276), *P* = 0.014, and 5045 (3370–9370) ng/mL versus 3244 (3020–3983), *P* < 0.001, resp.). All hormone levels and muscle fibre size (*n* = 12) remained stable throughout the study. Age-adjusted IGF-1 was associated with type 1 and 2 fibre sizes (*P* < 0.05).* Conclusion.* Patients' total testosterone values were normal due to markedly increased LH values, which suggest a compensated primary insufficiency of the testosterone producing Leydig cell. Even though testosterone values were normal, resistance training was not associated with muscle hypertrophy. This trial is registered with ISRCTN72099857.

## 1. Introduction

Endogenous testosterone and insulin-like growth factor 1 (IGF-1) are important factors in muscle anabolism in terms of stimulating muscle protein synthesis and inhibiting protein breakdown [1–3]. Thus, appropriate levels of anabolic hormones, as well as their function, are important for the avoidance of muscle atrophy, as well as the induction of muscle hypertrophy in relation to resistance training.

In male patients who undergo dialysis due to chronic kidney disease (CKD), serum testosterone levels are usually below, or in the low normal range, whilst luteinizing hormone (LH) may be elevated [4–9]. Changes in androgen synthesis and metabolism develop even with moderate reductions in renal function and may be the result of primary hypogonadism and/or disturbances in the hypothalamic-pituitary axis [6]. Uraemic toxins, comorbidities, and several drugs are believed to be contributory to the observed changes, but the exact mechanism remains unclear [6].

In addition to altered testosterone levels, the growth hormone action of muscle mRNA IGF-1 is also impaired [10]. Low levels of these anabolic hormones in patients undergoing dialysis affect muscle protein balance negatively leading to a reduction in muscle strength and impaired physical function [11, 12]. Whilst resistance training improves muscle strength in dialysis patients [13] the impact of testosterone levels on resistance training and muscle fibre size in these patients is generally unknown. We hypothesized that serum testosterone levels at baseline were decreased and associated with muscle fibre size in male patients undergoing dialysis. The aim of this study was to investigate association between resistance training and circulating levels of serum testosterone and IGF-1. The study was registered on http://www.controlled-trials.com/ under number ISRCTN72099857.

## 2. Materials and Methods

### 2.1. Participants

This study population comprised male patients from a recent study of effects of resistance training in dialysis patients conducted by us, where the primary outcomes were changes in quality of life, physical performance, and both muscle power and strength [13]. The men were included from three dialysis centres in or around the capital of Demark, to a control period of 16 weeks without any intervention followed by an intervention period of 16 weeks with resistance training. The inclusion criteria were age above 18 years, undergoing haemodialysis or peritoneal dialysis for more than three months, and the ability to participate in the training programme. Exclusion criteria were testosterone inhibiting medical treatment, insulin therapy, severe diabetic retinopathy, amputation of a lower limb, severe peripheral polyneuropathy, dementia, inability to speak Danish, and participation in other conflicting trials.

Data concerning renal disease and morbidity were obtained from case records, and levels of comorbidity were assessed using The Index of Coexistent Disease [14]. All tests were performed identically with regard to the dialysis procedure for each individual, before and after the control and training period to minimize interdialytic variation. Informed consent was obtained from all the patients and the local ethical committee approved this protocol (H-D-2008-124).

### 2.2. Intervention

The training programme has been described recently [13]. In brief, it consisted of supervised heavy load resistance training three times weekly for 16 weeks. The training began with 5 minutes of warm-up followed by up to 5 sets of leg press, leg extension, and leg curl. The rest period between each set was 60–90 seconds (the time between repetitions was not regulated). The programme was progressive and the load was increased according to increased muscle strength during the training period with a corresponding decrease in repetitions maximum from 15 to 6. Every set was performed to exhaustion.

### 2.3. Hormone Analyses

Blood samples were obtained in the morning hours after an overnight fast, a minimum of 18 hours after a training session. Serum was separated and kept frozen at −80° until analysis. Levels of testosterone, estradiol, LH, follicle-stimulating hormone (FSH), and sex hormone-binding globulin (SHBG) were analysed by fluoroimmunometric methods (Autodelfia, Wallac, Turku, Finland) and inhibin-B by an enzyme-linked immunosorbent assay (Inhibin-B GenII, Beckman Coulter, USA). IGF-1 and insulin-like growth factor-binding protein 3 (IGF-BP3) levels were determined by a chemiluminescence based assay (Immulite 2000, Siemens Healthcare Diagnostics, Tarrytown, USA).

The intra- and interassay coefficient of variation (CV) for measurement of testosterone were <2% and 8%, respectively. For estradiol <4% and 8%, for both LH and FSH <2% and 6%, SHBG <5% and <9%, inhibin-B <3% and <13%, IGF-1 <2% and <12%, and IGF-BP3 <4% and 11%, respectively. The limits of detection (LODs) were testosterone 0.3 nmol/L, estradiol 70 pmol/L, LH 0.05 IU/L, FSH 0.05 IU/L, SHBG 0.23 nmol/L, inhibin-B 3 pg/mL, IGF-1 20 ng/mL, and IGF-BP3 100 ng/mL.

Free testosterone levels were calculated from total testosterone, SHBG, and albumin as described previously [15]. For the reference population we used a fixed albumin level of 43.8 g/L when free testosterone was determined. Hormone ratios were calculated by simple division.

### 2.4. Muscle Fibre Analyses and ATPase Histochemistry

The muscle fibre analyses and ATPase histochemistry have been described in detail elsewhere [13]. Muscle biopsies were obtained from the mid-region of* m. vastus lateralis*, mounted with Tissue-Tek (Sakura Finetek, Zoeterwoude, The Netherlands), and immediately frozen in isopentane cooled in liquid nitrogen and stored at −80°C until analysis. Serial sections (10 *μ*m) from the muscle biopsy samples were cut and myofibrillar ATPase histochemistry was performed at pH 9.40 after preincubation at pH 4.37, 4.60, and 10.30 [16]. Computer image analysis was performed using an image analysis system (TEMA, Scan Beam ApS, Hadsund, Denmark). Fibre sizes were classified as major type 1 and type 2 [17]. Only truly horizontally cut fibres were included in the fibre size analyses.

### 2.5. Muscle Strength

Maximal voluntary knee extension was tested in an adjustable dynamometer chair (Good Strength, Metitur Ltd., Jyväskylä, Finland) at a knee angle of 60° from full extension. The patients were instructed to rapidly produce as much force as possible and hold it for 5 seconds. A minimum of three tests separated by 60 seconds of rest were conducted. For each subject the best performance with the highest value followed by a lower value was accepted as the result. The results were digitized into Newton (N) using the Good Strength software package (version 3.11. Metitur Ltd., Jyväskylä, Finland).

### 2.6. Blinding

The investigators who analysed the muscle morphology and hormone levels of the subjects were blinded to any other subject information, including the outcomes.

### 2.7. Statistical Analyses

Statistical analyses were carried out using IBM SPSS Statistics 19. The data distributions were tested using the Shapiro-Wilks test and Q-Q plots. Most residuals were found not to be normally distributed and statistical analyses were performed using nonparametric tests. The Wilcoxon Signed Ranks test was used to test for differences between baseline test and pretraining test (control period), between pretraining test and posttraining test (training period), and in-between periods. The Mann-Whitney test was used to compare the patients' data with data from the male reference population. The male reference population's hormone values were calculated using the patients' age as weights. The reference did not comprise LH, FSH, and testosterone/LH data for men older than 70 years and in these variables patients' data were compared with data for 70-year-old men from the reference population.

Binary correlations were tested using the Spearman test. Age adjustments were performed using linear regression analyses. In the linear regression analyses variables were included if *P* < 0.1 in binary correlations and variables were log-transformed if the residuals were not normally distributed.

Data are presented as the median (range), mean (standard deviation—SD), *t*, coefficients (95% confidence interval—CI), counts, or percentages. All tests were two-tailed and the level of significance was taken as *P* ≤ 0.05.

## 3. Results

**Figure 1:**
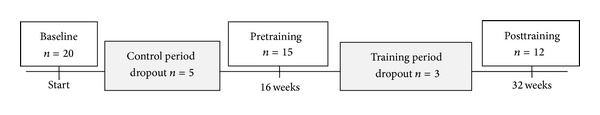
Study design and dropout.

Twenty patients were initially included in the study and 12 (haemodialysis, *n* = 11; peritoneal dialysis, *n* = 1) completed the intervention (see Figure 1). Dropout during the control period (*n* = 5) and during the training period (*n* = 3) was due to medical complications not related to the study. The patients who dropped out did not differ with regard to age, pretested comorbidity level, body mass index, or hormone profile compared to those who completed the study. The patients' characteristics are presented in Tables 1 and 2.

**Figure 2:**
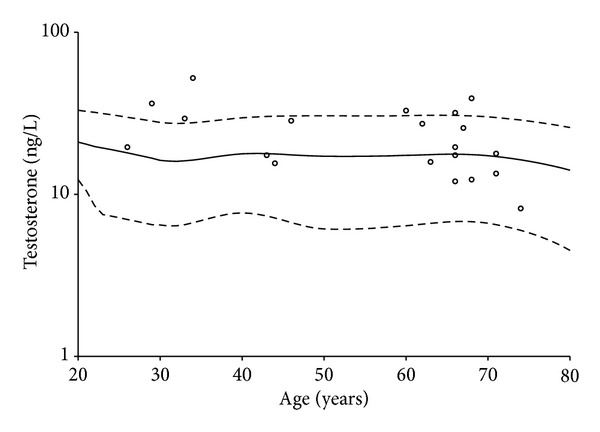
Testosterone levels plotted against the age of the patients. The dots represent male patients undergoing dialysis. Curves are mean ± 2SD for a male reference population.

**Figure 3:**
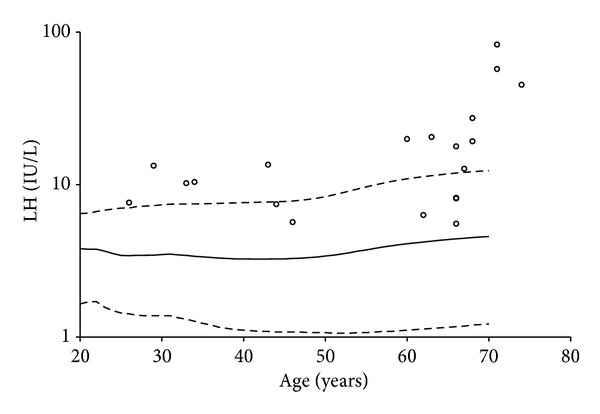
Luteinizing hormone levels plotted against the age of the patients. The dots represent male patients undergoing dialysis. Curves are mean ± 2SD for a male reference population.

Hormone profiles at baseline for the included patients are presented in Table 2 together with all the available data from the age-matched reference males. The patients' total testosterone was similar to the male reference and correlated negatively with age (*r* = −0.456, *P* = 0.04) (Figure 2). Free testosterone was elevated in the patients compared to the reference (Table 2). For all patients the LH values exceeded the age-matched means for the references (Figure 3), and within the patient group the correlation between LH and age was *r* = 0.555, *P* = 0.011. IGF-1 and IGF-BP3 were significantly higher in the patients compared to the reference group (Table 2), whereas IGF-1/IGF-BP3 did not differ. In correlations between the patients' testosterone and IGF-1, free testosterone was found to be positively associated with IGF-1 (*r* = 0.644, *P* = 0.002).

All hormones were found to be unchanged between baseline and the end of the training period. However, a significant change in total testosterone/LH was found when the delta values of the control period and the training period were compared (~−15% versus +17%, resp.) as presented in Table 3. Body mass index, haemoglobin, albumin, C-reactive protein, phosphate, and bicarbonate remained unchanged throughout the study.

Muscle fibre sizes remained unchanged during the study whereas muscle strength increased significantly by 19–25% during the training period (Table 4).

### 3.1. Testosterone and IGF-1 Correlated with Muscle Fibre Size

In unadjusted binary correlations between baseline tested anabolic hormones and muscle fibre size, free testosterone was found to be positively associated with type 2 muscle fibre size (*r* = 0.525, *P* = 0.025), whereas age-adjusted free testosterone was neither significantly associated with type 1 nor significantly associated with type 2 muscle fibre size (Table 5). Total testosterone was not associated with any muscle fibre size. Unadjusted IGF-1 was positively associated with type 1 fibre size (*r* = 0.626, *P* = 0.005) and type 2 fibre size (*r* = 0.598, *P* = 0.009). When IGF-1 was age-adjusted it remained associated with type 1 fibre size (*P* = 0.005) and type 2 fibre size (*P* = 0.012) (Table 5).

Prior to the training, hormone levels including total testosterone, free testosterone, testosterone/LH, and LH were not associated with individual changes in muscle fibre size during the training period (data not shown).

## 4. Discussion

To the best of our knowledge, this is the first study to investigate testosterone levels during a resistance training program in dialysis patients. The patients' serum testosterone levels were not different from those of a reference population whereas LH levels were markedly elevated, indicating a compensated primary Leydig cell insufficiency, a finding that has not been reported in recent studies but was reported in studies published in the 1970s [7]. Even though the testosterone values were within the normal range, the resistance training was not associated with muscle hypertrophy.

Our finding of normal total testosterone levels in the patient group is in contrast to the results of other studies that have detected relatively lower levels of testosterone in dialysis patients [4, 5, 8, 9]. Our study does not provide an explanation for this discrepancy, but we speculate that the results in our patient group may be indicative of a selected healthier subgroup of patients than previously published work. This speculative interpretation may, however, be supported by the patients' motivation to participate in a comprehensive training program. An increased LH level as a response to decreased Leydig cell capacity is an example of the classical endocrine feedback loop where the endocrine system tends to keep the peripheral hormones at a steady level. In our dialysis patients the decreased Leydig cell capacity was compensated by an increased LH stimulation. However, in general, some men go from being eugonadal (i.e., having normal testosterone levels on a background or normal LH levels) to having a compensated hypogonadism as our patients. Some patients may further develop an overt primary hypogonadism where an increased LH level is insufficient to compensate for the decreasing Leydig cell capacity as suggested by the European Male Aging Study [18]. The findings of reduced testosterone levels in previous studies of dialysis patients may reflect that these in general have reached the stage of primary hypogonadism. It is most likely the impaired kidney function in the dialysis patients that causes the decreasing Leydig cell capacity. To our knowledge it remains to be established to which degree testosterone deficient dialysis patients will benefit from a testosterone substitution therapy. However, it is tempting to speculate that they will benefit as many of the classical symptoms [18] between these two diseases overlap. Thus, our findings encourage a more detailed investigation focussing on this aspect.

Testosterone was unchanged after training and this was not surprising since the baseline values were relatively high. Testosterone/LH was also found to be unchanged after training. However, the statistical analyses showed a significant difference between delta values when comparing the control period with the training period. A slight decrease over the control period was followed by a slight increase over the training period in terms of the testosterone/LH value, resulting in a 32% net increase. This finding is most likely a chance finding, being the result of normal variation in testosterone. On the other hand, it cannot be excluded that a decrease in testosterone/LH in the patient sample was counteracted by a reverse through training. If this is indeed a true effect of training, then the underlying mechanism is not obvious. If the decrease in testosterone/LH was a true finding, it could be the result of improved metabolism leading to an improved testes function. However, further studies are needed before we are able to draw more definitive conclusions about such a relation.

The training intervention was not associated with muscle hypertrophy at the fibre level, which was an unexpected result. One would expect hypertrophy after a period of high load resistance training [19] especially when testosterone values are within a normal range. Indeed, the effect of resistance training on muscle mass is in general associated with testosterone level [20]. In healthy young men who conducted resistance training, an increase in muscle mass was significantly greater in subjects with normal testosterone compared to subjects with a suppressed testosterone level [20]. The anabolic effect of the training in our study may have been counteracted by impaired muscle protein synthesis or increased protein breakdown due to comorbidities [21, 22], as well as regular dialysis treatment [23]. Thus the significant effect on muscle strength observed in these patients may be primarily the result of neuromuscular improvements [24]. However, our study may have a relatively low statistical power, and one cannot ignore the possibility that the unchanged muscle fibre size that was observed could be the result of a type II error.

The relatively high levels of circulating IGF-1 and IGF-BP3 are in line with another study of resistance training in dialysis patients [25], although a decrease in IGF-1 following resistance training has also been reported [26]. Our finding of a positive association between IGF-1 and muscle fibre size at baseline is supported by MacDonald and colleagues who found a positive correlation between lean body mass and circulating IGF-1 in dialysis patients [27]. Whether circulating IGF-1 has a more significant role in muscle anabolism in patients undergoing dialysis compared to healthy subjects remains unknown.

The present study has important limitations. The sample size was small and heterogeneous in terms of age and dialysis duration. Furthermore, we did not measure body composition using scanning methods, which would have been interesting in addition to the data for muscle size at the fibre level. Finally, the study was limited by the nonrandomised design as a time series design was used. The design was used to elevate the number of patients, who received the intervention. However, a considerable strength of this study is that it presents data from a rigorous relatively long period of resistance training.

In conclusion, well treated male patients undergoing dialysis may counteract impaired Leydig cell function through elevated LH secretion. Even though testosterone and IGF-1 values were in or above the normal range for healthy individuals, the patients did not achieve muscle hypertrophy after a rigorous period of high load resistance training.

## Figures and Tables

**Table 1:** Characteristics of the participants.

Characteristic	(*n* = 20)
Age (years)	65 (26–74)
Dialysis modality (HD/PD)	19/1
Duration of chronic dialysis (years)	3.4 (1.1–15.0)
Comorbidities (score 0–3)	3 (1–3)
Body mass index (kg/m^2^)	25.6 (18.1–33.5)
Haemoglobin (mmol/L)	7.4 (6.4–9.9)
Albumin (g/L)	42.1 (37.4–49.8)
C-reactive protein (mg/L)	4.0 (0.6–23.0)
Phosphate (mmol/L)	1.8 (0.9–2.4)
Bicarbonate (mmol/L)	25 (17–33)
Primary renal disease	
Type 2 diabetes	1
Hypertension	3
Polycystic kidneys	4
Glomerulonephritis	7
Nephrosclerosis	1
Other/unknown	4

HD: haemodialysis; PD: peritoneal dialysis. Data are presented as median (range) or count.

**Table 2:** Hormone levels for the patients (*n* = 20) and for the age-matched male reference population.

Variables	All patients (*n* = 20)	Reference population	*P *
Total T (nmol/L)	19.5 (8.2–52.1)	17.6 (16.1–18.0)	0.174
Free T (pmol/L)	473 (174–1057)	289 (226–377)	0.004
IGF-1 (ng/mL)	203 (59–590)	151 (128–276)	0.014
IGF-BP3 (ng/mL)	5045 (3370–9370)	3244 (3020–3983)	<0.001
IGF-1/IGF-BP3	0.0443 (0.0158–0.0630)	0.04652 (0.0400–0.0694)	0.102
SHBG (nmol/L)	42.0 (8.0–132.0)	48.0 (32.9–59.4)	0.211
LH (U/L)	13.0 (5.5–82.8)	4.3 (3.3–4.6)	<0.001
FSH (U/L)	9.7 (1.7–86.1)	7.0 (3.3–7.5)	0.091
Total T/LH	2.1 (0.2–5.0)	4.1 (3.6–5.5)	<0.001
Estradiol (pmol/L)	105 (75–133)	117 (94–123)	0.096
Inhibin-B (pg/mL)	170 (1–403)	142 (134–165)	0.659
Inhibin-B/FSH	18.7 (0.1–235.7)	20.3 (17.9–49.0)	0.445

Data are presented as median (range). T: testosterone; SHBG: sex hormone-binding globulin.

**Table 3:** Hormone levels for those patients who completed the intervention (*n* = 12). The 16-week control period was measured from the baseline test to the pretraining test, and the 16-week training period was measured from the pretraining to the posttraining test.

Variables	Baseline (*n* = 12)	Pretraining (*n* = 12)	Posttraining (*n* = 12)	*P* control period	*P* training period	*P* between periods
Total T (nmol/L)	21.7 (8.2–52.1)	18.7 (7.8–54.7)	19.8 (7.2–57.7)	0.695	0.326	0.099
Free T (pmol/L)	485 (141–1057)	474 (177–820)	524 (240–776)	0.695	0.530	0.347
IGF-1 (ng/mL)	203 (138–590)	246 (108–811)	201 (113–606)	0.346	0.272	0.388
IGF-BP3 (ng/mL)	4905 (3600–9370)	4740 (3610–9760)	4525 (3550–8900)	0.754	0.844	0.937
IGF-1/IGF-BP3	0.0443 (0.0313–0.0630)	0.0461 (0.0262–0.0831)	0.0423 (0.0267–0.0895)	0.433	0.182	0.347
SHBG (nmol/L)	42.5 (13.0–66.0)	35.0 (11.0–80.0)	38.5 (8.0–83.0)	0.235	0.366	0.239
LH (U/L)	13.4 (5.5–57.2)	15.8 (5.6–58.7)	14.4 (5.5–52.0)	1.000	0.424	0.477
FSH (U/L)	10.5 (4.1–86.1)	9.7 (3.9–84.0)	8.9 (3.3–83.7)	1.000	0.272	1.000
Total T/LH	1.83 (0.18–5.01)	1.55 (0.24–2.86)	1.82 (0.34–3.70)	0.084	0.110	0.041
Estradiol (pmol/L)	101 (75–133)	104 (70–113)	101 (68–125)	0.432	0.694	0.638
Inhibin-B (pg/mL)	170 (1–342)	162 (1–380)	172 (1–355)	0.929	0.721	0.575
Inhibin-B/FSH	15.35 (0.01–83.82)	15.77 (0.01–88.99)	20.31 (0.01–107.90)	0.937	0.136	0.209

Data are presented as median (range). T: testosterone; SHBG: sex hormone-binding globulin; DHEA: dehydroepiandrosterone; AMH: anti-Müllerian hormone.

**Table 4:** Muscle fibre size and muscle strength for those patients who completed the intervention (*n* = 12). The control period was between the baseline and pretraining test. The training period was between the pretraining and the posttraining test.

Variables	Baseline	Pretraining	Posttraining
Muscle fibre size			
Type 1 (*µ*m^2^)	4896 (3138–8453)	4760 (2853–7891)	4730 (2273–9204)
Type 2 (*µ*m^2^)	3485 (2778–6067)	3246 (2284–5336)	3832 (2829–6953)
Knee extension strength			
Right (N)	349 (195–511)	349 (200–583)	460 (258–695)***
Left (N)	299 (216–595)	341 (193–558)	400 (238–657)**

Data are presented as median (range). N: Newton. Between pretraining and posttraining **P < 0.010; ***P < 0.005.

**Table 5:** Age adjusted free testosterone and IGF-1 correlations with muscle fibre size (dependent variables).

Muscle fibre size	Free testosterone (pmol/L)			IGF-1 (ng/mL)		
	*t *	Coefficient* (95% CI)	*P *	*t *	Coefficient* (95% CI)	*P *
Type 1 (*µ*m^2^)	1.61	0.00 (0.00-0.00)	0.128	3.29	0.35 (0.12–0.58)	0.005
Type 2 (*µ*m^2^)	1.83	1.596 (−0.259–3.450)	0.087	2.84	2348 (586–4110)	0.012

*Unadjusted. CI: confidence interval.
